# Monthly Summary

**Published:** 1869-01

**Authors:** 


### MONTHLY SUMMARY.

*Cancer of Antrum; Extirpation of Upper Jaw.* (Service of Dr. Derby.)—

S. P., a widow, aged 50, a washerwoman by occupation, had complained for six months of distressing neuralgia confined to the right side of the head and face, and especially severe about the right ear. Medication failed to relieve it. Shortly after her entrance to the hospital, a slight protuberance in the palate was discovered, situated on the right side just within the alveolar border. The right nostril had been obstructed during the previous month. The two sides of the face were symmetrical, but a tender point was noticed just outside and above the right ala. No tumor could be felt in the posterior palatine region, and the posterior nares were free. The flow of saliva was unnaturally profuse. There was no epiphora, and there had been no epistaxis.

During the three weeks succeeding her entrance, attention was devoted to alleviating the neuralgic symptoms, and to the support of the system in general. The tumor in the palate enlarged steadily, and became more tender. All the symptoms indicated malignant disease of the antrum, and the removal of the bone was determined upon.

A primary incision was made, upward, along the commissure of the upper lip, and outward around the ala, and, again, upward, to within half an inch of the inner canthus. The flaps were dissected on either side, so as to expose, on one side, the lower meatus, and on the other, the body of the upper jaw as far as its articulation with the malar bone. The anterior wall of the antrum, thus exposed, presented a nodule as large as a filbert. With a Hey's saw, a section of the bone was made, extending from the posterior tuberosity, forward, to the middle meatus of the nose. The palate process was divided with bone forceps, and the soft tissues being divided with a scalpel in the line of articulation with the palate bone, the section of bone was torn away by means of "lion" forceps, and the extent of the disease was thus exposed. The antrum was filled with soft cancerous growth, and the turbinated bones were also involved. The disease was found to extend upward towards the frontal sinuses, outward under the orbital floor, and backward to the pterygoid processes. It was deemed advisable, on this account, to to remove portions of the malar and nasal bones. The primary incision was accordingly met at its termination near the eye by another at right angles, extending, just below the lower lid, outward four inches. This flap having been lifted, section of the zygoma and of the orbital process was made by a saw. The bone was separated from its attachment, not without some difficulty, the progress of the disease having rendered the bony tissue fragile. The hæmorrhage was so inconsiderable as to require no ligatures. The flaps were at once apposed and held by silk sutures. There was no noteworthy shock. The external wound healed with very satisfactory rapidity, union by the first intention being secured, except at one point. The cavity of the wound, internally, was syringed frequently with diluted liquor soda chlorinatæ, and granulations were healthy. On the fourth day, the sutures were removed. There were still some of the neuralgic pains remaining, confined chiefly to the supraorbital and temporal regions. The external deformity was notably slight, although articulation was much impaired.

In the third week after the operation, œdema of the lower lid of the affected side manifested itself, and was persistent. In the cavity of the mouth, along the edge of the hard palate, the granulations had assumed an exuberant and anæmic appearance, much resembling the original diseased growth, and indicating its recurrence. The neuralgic pains continued, requiring the administration of morphia. At the end of the fourth week she was discharged, relief from her former symptoms having been obtained only in moderate degree—*Boston Med. and Surg. Journal.*

*Nasal Therapeutics.*—At a recent meeting of the Liverpool Medical Institution, reported in the *British Medical Journal*, Dr. Banks, one of the members, made the following extremely practical remarks on the Applications of Remedies to the Nostrils and Larynx:—

"Weber, of Leipzic, discovered fifty years ago that when a column of water was passed along one nostril, when it touches the soft palate, it causes it to rise so as to shut off the nasal from the pharyngeal cavity, so that the fluid is compelled to return through the other nostril. Weber, of Halle, first put the principle into practice; and in 1864, Dr. Thudichum invented an instrument, and published some papers on the subject, The points to be attended to in using the instrument were the following: 1, the nozzle should fit the nostril accurately; 2, in children and nervous people, the full stream should not be turned on at once; it should be allowed to pass in gently at first, and then gradually increase in volume and force; 3, the current should be reversed occasionally. Cold water is irritating; and, therefore, tepid water, or a solution of an ounce of salt in a pint of water, may be used, followed by a deodorizer, as Condy's fluid, liquor carbonis detergens, or especially carbolic acid, and afterwards a stimulant astringent, as alum (one drachm to the pint,) sulphate of zinc or copper (from ten to thirty grains to the pint,) etc. The solution should not be too strong at first. The instrument was also useful in some surgical accidents, such as a foreign body in the nostril and severe epistaxis, when some dilute hæmostatic should be employed. Dr. Skinner had been practically making use of this principle before Dr. Thudichum's paper appeared, the instrument employed being a Higginson's syringe. The author had found Mr. Byrant's mode of treating nasal polypi, by blowing tannin into the nostrils through a quill, very satisfactory in some cases, especially soft and gelatinous polypi. Another troublesome affection—a chronically swollen and thickened condition of the nasal and palatine mucous membrane—was benefited by the administration of iodide or bromide of potassium; but local astringents were also useful, and were best applied by means of the spray producer. The best applications were, glycerine of tannic acid (one scruple to one ounce of water,) or a solution of iodine, with a small quantity of carbolic acid. Speaking of affections of the throat, the author observed, that of the various instruments devised to bring remedies into contact with the air-passages, the spray producer was the best. Its use was very great in chronic laryngeal affections, and also in many acute affections, as putrid sore throat and scarlatinal cynanche, diptheria. The spray producer could not be employed with very potent remedies, such as strong solution of nitrate of silver. A piece of whalebone, bent at an obtuse angle near the end, and having a brush (better than a sponge) attached to it, was the best instrument for applying these. Care and dexterity are requisites in using it."—*Med. and Surg. Reporter.*

---

*Dissections.*—A knowledge of anatomy is necessary alike to the physician and to the surgeon. Without it they are both the merest blunderers, practicing with no fixed principles, and affecting cure solely by accident, or through the benignant ministrations of nature. It is, in fact, the foundation stone upon which the whole superstructure of medicine is reared—the source of all that is certain and scientific in the healing art.

This information can be obtained in but one way. Plates, models, and illustrations, howevor well devised or skillfully executed, are only adjuvants in the study of anatomy. A thorough acquaintance with the structure, relations and functions of the component elements of the human organism is acquired only by *dissection*—comes at the point of the scalpel, or not at all.

Dissections, are, of course, impossible without *subjects.* Unless anatomical material can be had in sufficient quantity, the science of anatomy itself must remain a "terra-incognita"—an impenetrable mystery—forever. The proper steps for supplying the demands of the dissecting room are thus necessitated and legitimatised upon the plainest principles of logic and common sense.

And yet intelligent men, whose appreciation of professional acquirements is illustrated by daily appeals to the skill of physicians, are "shocked and outraged" at the idea of consecrating the bodies of the dead to the purposes of science.

The law, too, with strange inconsistency, demands of every medical man a thorough acquaintance with medicine in all of its departments, and at the same time makes the only possible means whereby the most essential portion of such knowledge can be acquired, an infamous crime.

Thus it is that society and the courts unite in the paradox of simultaneously exacting and precluding knowledge—of reprobating and enforcing unskillfulness—of fostering and yet preventing such advances in science as are demanded by the highest and dearest interests of the race.

There is neither sense nor justice in contradictions like these. The prejudices which involve such palpable and illogical inconsistencies, are unworthy of intelligent and honorable men. The statutes which thus place the law in direct antagonism to itself. as well as to reason and humanity, deserve no place in the code of an enlightened and christian people. A healthy public sentiment should control the one, and a wise legislation abrogate the other. —*Balto. Med. Bulletin.*

---

*Man's Antiquity in America.*—At the meeting of the American Association for the advancement of Science, recently held in the city of Chicago, Colonel Whittlesey maintained that four American races preceded the red man. First, the mound-builders; [illegible]

[illegible] statuette of a captive with his hands bound behind him, both from Peru, and evidently of extreme antiquity. The recent explorations of Mr E. G. Squiers have renewed some old theories as to a connection in origin between the earliest inhabitants of America and those of the Oriental countries. These statements remain, however, as theories only, and do not deserve to be classed with ascertained scientific facts.—*Med. & Surg. Reporter.*

